# Ophthalmic Intervention of Naringenin Decreases Vascular Endothelial Growth Factor by Counteracting Oxidative Stress and Cellular Damage in In Vivo Zebrafish

**DOI:** 10.3390/molecules28145350

**Published:** 2023-07-12

**Authors:** Gokul Sudhakaran, Abhirami Chandran, A. R. Sreekutty, S. Madesh, Raman Pachaiappan, Bader O. Almutairi, Selvaraj Arokiyaraj, Zulhisyam Abdul Kari, Guillermo Tellez-Isaias, Ajay Guru, Jesu Arockiaraj

**Affiliations:** 1 Toxicology and Pharmacology Laboratory, Department of Biotechnology, Faculty of Science and Humanities, SRM Institute of Science and Technology, Kattankulathur 603203, India; gokulsudhakaran98@gmail.com (G.S.); abhiramichandran0126@gmail.com (A.C.); sreekuttykavalam2000@gmail.com (A.R.S.); ms4329@srmist.edu.in (S.M.); 2 Department of Biotechnology, School of Bioengineering, College of Engineering and Technology, SRM Institute of Science and Technology, Kattankulathur 603203, India; pachaiar@srmist.edu.in; 3 Department of Zoology, College of Science, King Saud University, P.O. Box 2455, Riyadh 11451, Saudi Arabia; bomotairi@ksu.edu.sa; 4 Department of Food Science & Biotechnology, Sejong University, Seoul 05006, Republic of Korea; arokiyaraj16@sejong.ac.kr; 5 Department of Agricultural Sciences, Faculty of Agro-Based Industry, Universiti Malaysia Kelantan, Jeli Campus, Jeli 17600, Malaysia; zulhisyam.a@umk.edu.my; 6 Advanced Livestock and Aquaculture Research Group, Faculty of Agro-Based Industry, Universiti Malaysia Kelantan, Jeli Campus, Jeli 17600, Malaysia; 7 Department of Poultry Science, University of Arkansas, Fayetteville, AR 72701, USA; gtellez@uark.edu; 8 Department of Cardiology, Saveetha Dental College and Hospitals, SIMATS, Chennai 600077, India

**Keywords:** diabetic retinopathy, naringenin, vascular endothelial growth factor, oxidative stress, zebrafish

## Abstract

Diabetes Mellitus is a metabolic disease that leads to microvascular complications like Diabetic retinopathy (DR), a major cause of blindness worldwide. Current medications for DR are expensive and report multiple side effects; therefore, an alternative medication that alleviates the disease condition is required. An interventional approach targeting the vascular endothelial growth factor (VEGF) remains a treatment strategy for DR. Anti-VEGF medicines are being investigated as the main therapy for managing vision-threatening complications of DR, such as diabetic macular oedema. Therefore, this study investigated the effect of flavonoid naringenin (NG) from citrus fruits on inhibiting early DR in zebrafish. When exposed to 130 mM glucose, the zebrafish larvae developed a hyperglycaemic condition accompanied by oxidative stress, cellular damage, and lipid peroxidation. Similarly, when adult zebrafish were exposed to 4% Glucose, high glucose levels were observed in the ocular region and massive destruction in the retinal membrane. High glucose upregulated the expression of VEGF. In comparison, the co-exposure to NG inhibited oxidative stress and cellular damage and restored the glutathione levels in the ocular region of the zebrafish larvae. NG regressed the glucose levels and cellular damage along with an inhibition of macular degeneration in the retina of adult zebrafish and normalized the overexpression of VEGF as a promising strategy for treating DR. Therefore, intervention of NG could alleviate the domestication of alternative medicine in ophthalmic research.

## 1. Introduction

Diabetic retinopathy (DR) is one of the prevalent microvascular complications that occurs in individuals with hyperglycaemia and leads to vision loss and subsequent blindness [1]. The extent of glycaemic control and the age of diabetes significantly influences the possibility of experiencing DR. Recent studies show that about 100 million people worldwide are affected by DR, which is predicted to become a growing health problem [2].

Neural retina dysfunction and cell degeneration coexist with preeminent micro-vascular vicissitudes, compromised vessel integrity, decreased perfusion, and neovascularization. Vascular changes, like loss of endothelial cells, often secondary to damage; reduced capillary blood flow; and microaneurysms, are involved in the initial stage of DR [3]. The core remedies for DR that imperil or harm your vision are laser treatment, eye injection, and eye surgery. The fundamental medications are aflibercept (Eylea) and ranibizumab (Lucentis). Vitreoretinal surgery has been used to eliminate the vitreous humour from the eye. These medications may create side effects such as blurred vision, eye bleeding, increased sensitivity, and aching [4].

Due to the high side effects of the treatments, multiple classes of molecules have been explored for their further potential to reduce the risk of retinopathy with pharmacological treatment. Plants have been the most treasured source of remedies [5]. The active chemical elements of plants are naturally occurring compounds with biological activities. Neovascularisation and macular oedema have been treated using therapeutic approaches that aim for a vascular endothelial growth factor (VEGF) [6]. Hence, natural agents of dietary supplements that suppress the VEGF signalling pathway could be a promising strategy to inhibit early DR [7].

Citrus flavonoids have been proven to protect against various diseases, including diabetes and its comorbidities, by reducing inflammation and oxidative stress [8]. Naringenin (NG) is one of the flavanones abundant in citrus fruits such as tomatoes (*Lycopersicon esculentum*) and oranges (*Citrus sinensis*) [9,10,11]. NG is considered one of the main bioactive flavanones, and the total concentration of NG in human plasma is up to 11.04 μM after single oral administration [12,13]. Therefore, in this study, we investigate the ability of NG to inhibit oxidative stress, cellular damage, and lipid peroxidation in a developing zebrafish and prevent early DR by inhibiting VEGF in a hyperglycaemic adult zebrafish.

## 2. Results

### 2.1. High Glucose Treatment Induced Survival and Hatching Rate of Zebrafish Larvae

**Figure 1:**
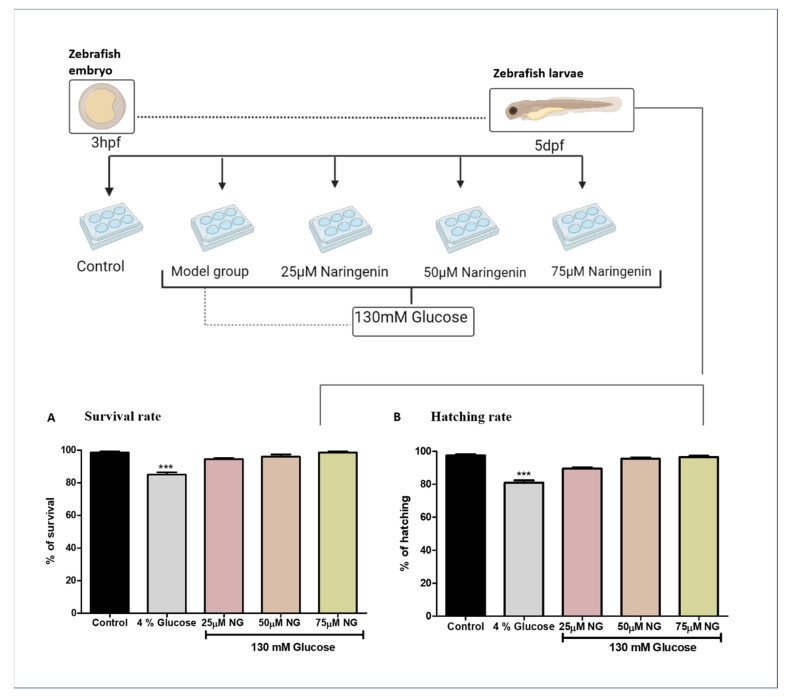
**Treatment paradigm for the exposure of NG to zebrafish embryos.** Effect of NG in co-exposure with 130 mM glucose on (**A**) survival and (**B**) hatching rates in zebrafish larvae. Experiments were performed in triplicate, and the data were expressed as mean ± SD. *** represents the significant difference *p* < 0.001 compared to the control.

The zebrafish embryos exhibited no significant mortality and hatching rates in the NG treatment group compared to the control group. Comparatively, high glucose treatments were toxic to the model group because of the prolonged exposure of glucose to the zebrafish. The high glucose treatment groups observed a reduction in the mortality and hatching rate (Figure 1).

### 2.2. Total Glucose Levels in Zebrafish Larvae Post High Glucose Exposure

**Figure 2:**
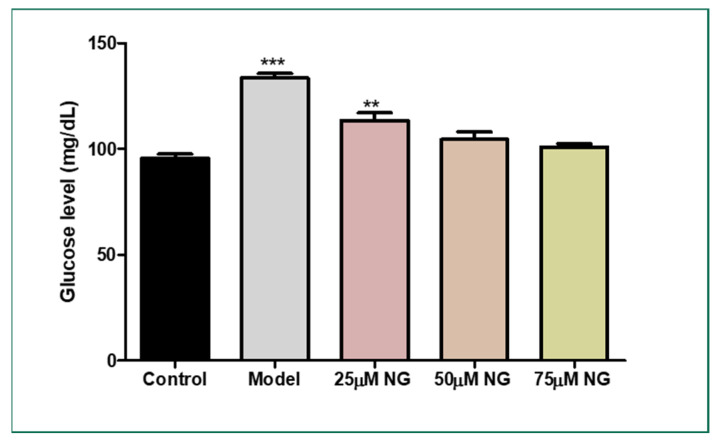
Effect of NG in co-exposure with 130 mM glucose and glucose level estimation in zebrafish larvae. Experiments were performed in triplicate, and the data were expressed as mean ± SD. ** represents the significant difference *p* < 0.01 and *** represents *p* < 0.001.

The total glucose levels were estimated in the pooled supernatant of the zebrafish larvae. Higher glucose levels were observed in the model group (133.5 mg/dL) compared to the control group (95 mg/dL). NG at a concentration of 75 µM almost reversed the condition (102.5 mg/dL), almost similar to the control group (Figure 2).

### 2.3. High Glucose-Induced ROS Generations

**Figure 3:**
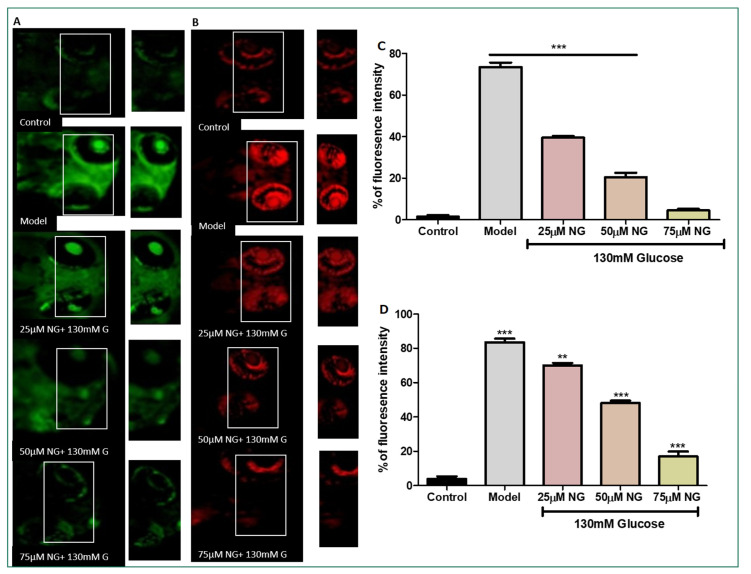
(**A**). Effect of NG in co-exposure with 130 mM glucose-induced intracellular ROS in the ocular region of the zebrafish larvae using DCFDA fluorescent probe. The retina has been highlighted in the white box. The fluorescence image was captured using a fluorescence microscope. (**C**) Experiments were performed in triplicate, and the data were expressed as mean ± SD. *** represents the statistical significance at *p* < 0.001 compared to the control. (**B**). Effect of NG in co-exposure with 130 mM glucose-induced intracellular O^−^_2_ in the ocular region of zebrafish larvae using NDA fluorescent probe. The ocular region has been highlighted in the white box. The fluorescence image was captured using a fluorescence microscope. (**D**) Experiments were performed in triplicate, and the data were expressed as mean ± SD. ** represents the significant difference *p* < 0.01 and *** represents *p* < 0.001. (**C**,**D**) Fluorescent intensity of the zebrafish larvae calculated using Image J software.

DCFHDA fluorescent dye was used to detect the production of ROS in the ocular region as an impact of high glucose treatment. In zebrafish larvae treated with high glucose, ROS levels increased to 75%. However, NG (25, 50, and 75 μM) and treated groups showed a dose-dependent reduction in ROS levels measured using fluorescent intensity (39.5%, 20.5%, and 4%). NG reduced intracellular ROS levels in a concentration-dependent manner (Figure 3A).

### 2.4. High Glucose-Induced Superoxide Anion Production

Superoxide anion indicator DHE was used to assess the number of superoxide anions generated. Increased superoxide anion levels and oxidative stress were indicated by an increase in the percentage of cells expressing high levels of DHE. The model group exhibited a significant increase in the intensity of DHE (83%), whereas, in comparison to the model group, the ROS levels were diminished significantly in the NG-treated group (25, 50, and 75 μM) in a dose-dependent manner (70%, 48% and 17%) (Figure 3B).

### 2.5. High Levels of ROS Deplete the Total GSH Levels

**Figure 4:**
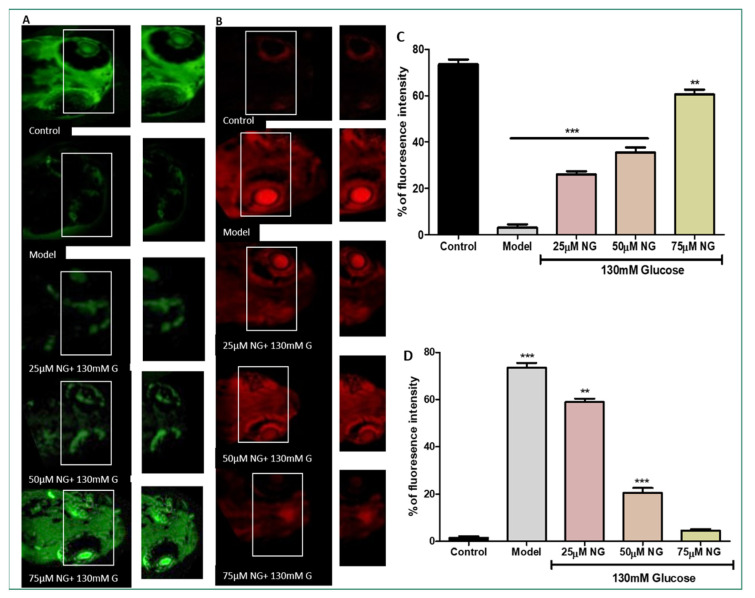
(**A**) Effect of NG in co-exposure with 130 mM induced glutathione depletion in the ocular region of zebrafish larvae using NDA fluorescent probe. The ocular region has been highlighted in the white box. The fluorescence image was captured using a fluorescence microscope. (**C**) Experiments were performed in triplicate, and the data were expressed as mean ± SD. ** represents the significant difference *p* < 0.01 and *** represents *p* < 0.001. (**B**) Effect of NG in co-exposure with 130 mM glucose on intracellular apoptosis and cellular damage in zebrafish larvae using acridine orange staining. The ocular region has been highlighted in the white box. The fluorescence image was captured using a fluorescence microscope. (**D**) Data were expressed as mean ± SD of three independent experiments. ** represents the significant difference *p* < 0.01 and *** represents *p* < 0.001. (**C**,**D**) Fluorescent intensity of the zebrafish larvae calculated using Image J software.

NDA was used to measure the total GSH levels in the zebrafish larvae. Compared to the model group, a considerable increase in GSH levels was observed in zebrafish larvae exposed to 25–75 μM NG. At a minimal concentration of NG 25 μM, 25% fluorescent intensity, and a higher concentration of NG 75 μM, 60% fluorescent intensity was observed. A higher concentration of NG revealed higher fluorescence intensity. Compared to the model group, the treated group’s GSH levels were significantly higher, almost similar to the control group (Figure 4A).

### 2.6. High Levels of ROS-Induced Cellular Damage and Lipoxidation

AO staining distinguishes apoptotic cells [14]. Therefore, the results from AO staining showed that the model group had higher prominent apoptotic cells or cells underdoing cellular damage. Apoptosis was also noticeably increased in the ocular region of the model group (75%). In contrast, the frequency of apoptotic cells developed in the ocular region in the NG-treated groups was considerably less. At a minimal NG concentration of 25 μM, 57% was observed, and, at a higher NG concentration of 75 μM, 5% of poor fluorescence intensity was observed, indicating the inhibition of cellular damage. Therefore, NG could prevent cell death caused by high glucose treatment (Figure 4B).

**Figure 5:**
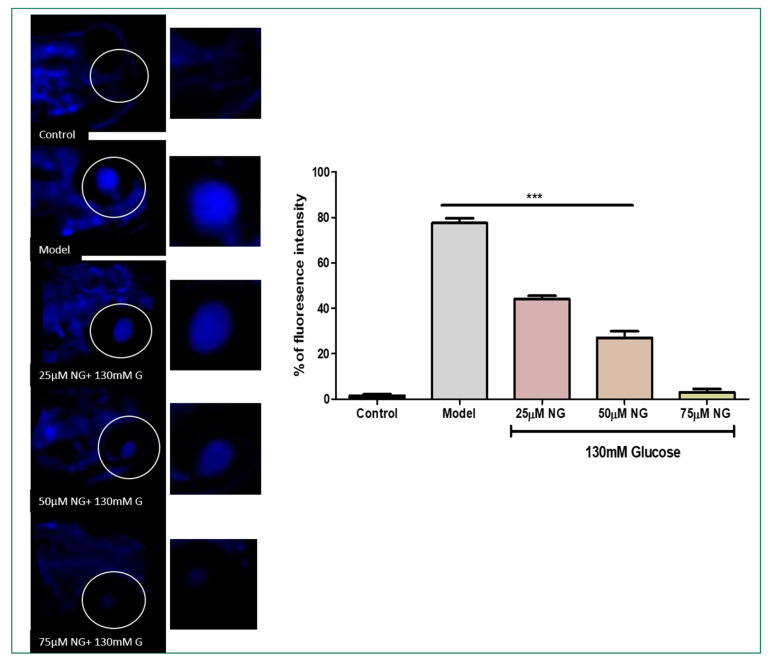
Effect of NG in co-exposure 130 mM glucose on intracellular lipid peroxidation in zebrafish larvae using DPPP staining. The ocular region has been highlighted. The fluorescence image was captured using a fluorescence microscope. Data were expressed as mean ± SD of three independent experiments. *** represents the significant difference *p* < 0.001.

The reactive intermediates generated by oxidative stress can change membrane bilayers and promote lipid peroxidation. The DPPP dye was used on zebrafish larvae to access the cell membrane lipid peroxidation induced by high glucose treatment. The model group exhibited a significant increase in the intensity of DPPP (78%). At a minimum concentration of 25 μM, NG exhibited 41%; at a concentration of 75 μM, NG exhibited the lowest fluorescent intensity 7%. The results demonstrated that NG effectively prevented the membrane damage induced by high glucose treatment (Figure 5).

### 2.7. High Glucose Exposure to Adult Zebrafish and Total Glucose Levels

**Figure 6:**
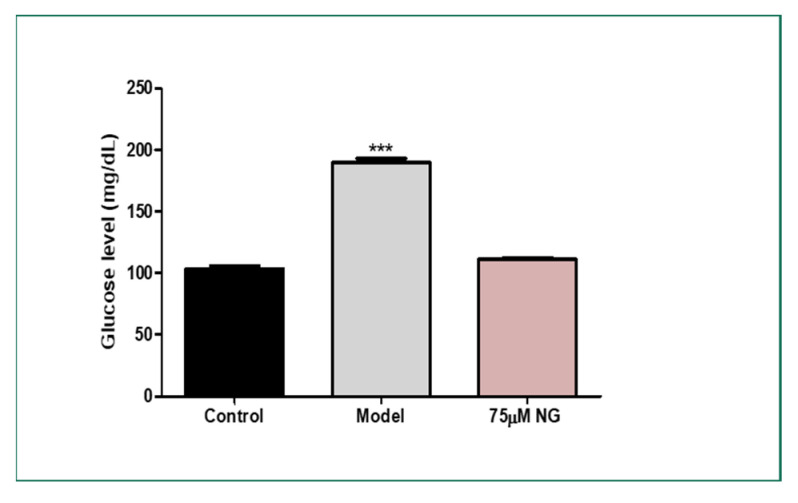
Effect of NG in co-exposure with 4% glucose and glucose level estimation in adult wild-type zebrafish. Experiments were performed in triplicate, and the data were expressed as mean ± SD. *** represents the statistical significance at *p* < 0.001 compared to the control.

The total glucose levels were estimated in the pooled mixture of the zebrafish. High glucose levels were observed in the model group (189 mg/dL) compared to the control group (103 mg/dL). At the same time, NG at a concentration of 75 μM exhibited (120 mg/dL). Therefore, NG could attenuate hyperglycaemic conditions in the zebrafish larvae and the adult zebrafish (Figure 6).

### 2.8. High Glucose-Induced Morphological Alterations in the Ocular Region and VEGF Regulation

**Figure 7:**
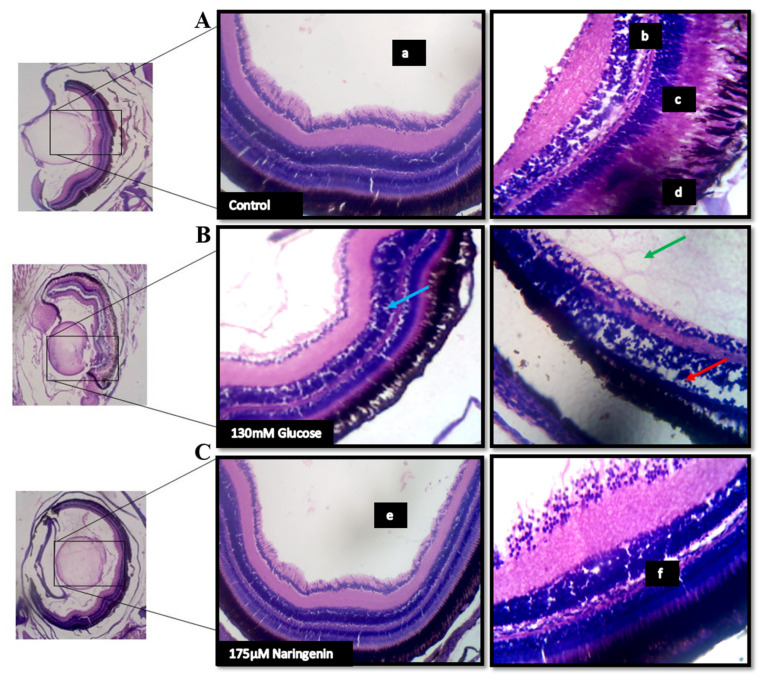
The histopathological examination of zebrafish eye (Lateral section): (**A**) Control; (**B**) Model group (Glucose treated); (**C**) Treatment group; (a) lens; (b) retinal cells; (c) rods and cones; (d) Pigment epithelium in the control group. The red arrow indicates a disruption in the retinal cell layer; the blue arrow indicates the vacuole formation and necrosis in the rods and cones layer; the green arrow indicates the massive destruction in the lens. (e) lens and (f) the layers of the retina being normalized after the treatment.

**Figure 8:**
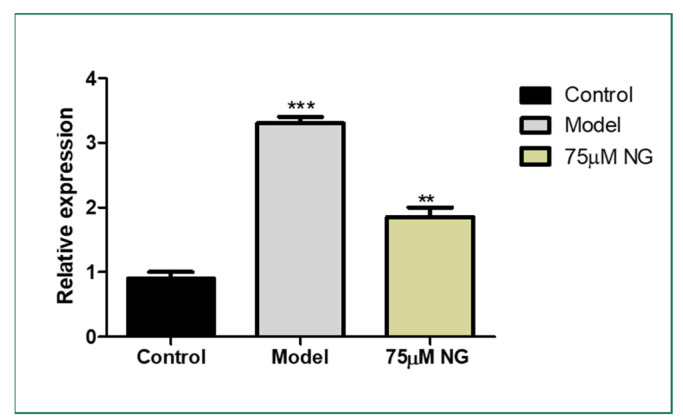
Effect of 75 μM NG on the mRNA expression of VEGF. Data were expressed as mean + SD of three independent experiments. ** represents the significant difference *p* < 0.01 and *** represents *p* < 0.001.

The lateral sectioning of the zebrafish revealed that high glucose treatment disrupted the retinal cell layers and induced necrosis in the rods and cones, along with vacuole formation. Massive destruction in the retina was observed. Whereas, in the control group, no histological alterations were observed, and the NG-treated zebrafish exhibited minimal damage to the retina by inhibiting necrosis and macular degeneration (Figure 7). The results also correlated with the gene expression studies, where VEGF was upregulated in the model group compared to the control group, whereas the treatment of NG regressed the expression of VEGF, similar to the control group (Figure 8).

## 3. Materials and Methods

### 3.1. Culture and Maintenance of Zebrafish

Adult wild-type zebrafish were acquired from NSK Aquarium, Kolathur, Tamil Nadu. The wild-type zebrafish adults were maintained at 26–28.5 °C in a 10 L glass tank. Live brine shrimp (*Artemia salina*) nauplii are fed to the zebrafish. The fish were utilized for breeding to produce embryos after acclimating for a month. Breeding groups were separated in spawning tanks with a male-to-female ratio of 2:1 and mesh at the bottom to prevent adult fish from devouring the eggs. The egg yolk was administered to the larvae during the experiment [15]. The zebrafish were handled carefully per the Institute Animal Handling Procedure and Ethical Approval and Clearance (No. SAF/IAEC/211215/004).

### 3.2. Glucose Treatment for the Induction of DR in Developing Embryos

About 30 zebrafish embryos in each group were exposed to a high concentration of glucose (130 mM) and varying concentrations of NG (commercially procured from Sigma-Aldrich, St. Louis, MO, USA) in embryonic medium (E3) for 3 h post-fertilization (hpf) to 5 days post fertilization (dpf). The control group had no exposure to NG and glucose. The zebrafish larvae post 6 days of co-exposure with NG and 130 mM Glucose were analysed for survival and glucose levels and taken for further experiments [3].

### 3.3. Estimation of Glucose Levels in the Zebrafish Larvae

The relative glucose content was measured in the zebrafish larvae. Twenty larvae from each group were pooled and homogenized in a 1.5 mL centrifuge tube. The glucose levels were estimated using the glucose estimation kit (Robonik, Ambernath, India). The glucose levels in each sample were calculated [16,17].

### 3.4. ROS Quantification Using Di-Chloro-Di-Hydro-Fluorescein Diacetate (DCFH-DA)

The production of intracellular ROS in the ocular region of zebrafish larvae was detected by the oxidation-sensitive fluorescent probe dye DCFH-DA using a previously established protocol with minor modifications [18,19]. The 5 dpf larvae were added to a 6-well plate containing E3 medium following the Glucose and NG exposure. DCFDA (20 μg/mL) was added to stain larvae for 1 h. During the post-incubation period, the larvae were rinsed with Phosphate-buffered saline (PBS) and were observed under a fluorescent microscope (Olympus, Tokyo, Japan). The fluorescent intensity is proportional to the level of ROS in the ocular region. The fluorescence intensity was assessed using Image J software (version 1.49) NIH, USA.

### 3.5. Superoxide Anion Radical Staining Using Dihydroethidium (DHE)

The production of Superoxide anion radicals in the ocular region of zebrafish larvae was detected using a DHE stain using a previously established protocol with minor modifications [20,21]. The larvae were treated with (20 μg/mL) DHE probes for 30 min at 28 °C. During the post-incubation period, the larvae were rinsed with PBS and were observed under a fluorescent microscope (Olympus, Tokyo, Japan). The superoxide anion levels were quantified by their fluorescence intensity. The fluorescence intensity was assessed using Image J software.

### 3.6. Total Glutathione (GSH) Level Estimation Using Naphthalene Di-Carboxaldehyde (NDA) 

The total GSH levels in the zebrafish larvae were measured using an NDA stain [17]. The zebrafish larvae were treated with 50 µM NDA and incubated at 37 °C for 30 min. After incubation with the fluorescent probe, the larvae were rinsed using PBS and observed under a fluorescent microscope (Olympus, Tokyo, Japan). The total GSH levels were quantified by their fluorescence intensity. The fluorescence intensity was assessed using Image J software.

### 3.7. Cellular Damage Staining Using Acridine Orange (AO)

AO is a specific nucleic acid dye that measures the ocular region’s cellular damage. Live larvae were treated with the nucleic-acid-selective metachromatic stain AO to determine cellular damage [19,22]. AO primarily stains cells with decreased plasma membrane potential; it predominantly detects neurotic or late apoptotic cells. The medium for the post-treated larvae was replaced, treated with an AO solution (60 μM), and then incubated for 30 min at 28.5 °C in the dark. Post-incubation with the fluorescent dye, larvae were rinsed with PBS to remove excess stain and were observed under a fluorescent microscope (Olympus, Tokyo, Japan). The cellular damage was quantified by its fluorescence intensity. The fluorescence intensity was assessed using Image J software.

### 3.8. Lipoxidation Staining Using 1,3-Bis (Di-Phenyl-Phosphino) Propane (DPPP)

The lipid peroxidation levels were stained in the retinal region of the zebrafish larvae using a DPPP stain [23]. The medium for the post-treated larvae was replaced, treated with DPPP stain (20 μM), and then incubated for 30 min at 37 °C. Post-incubation with the fluorescent dye, larvae were rinsed with PBS to remove excess stain and were observed under a fluorescent microscope (Olympus, Tokyo, Japan). The lipoxidation levels were quantified by their fluorescence intensity. The fluorescence intensity was assessed using Image J software [24,25,26,27].

### 3.9. Glucose Treatment in Wild-Type Zebrafish to Induce Hyperglycaemic Condition

Wild-type zebrafish adults, independent of sex, were intra-peritoneally injected (20 µL) with NG and were incubated in an alternating high-glucose medium, up to 10% for two weeks to induce a hyper-glycaemic condition followed by a previously established protocol with minor modifications [3]. The medium was changed once in two days. The lens of the zebrafish was isolated, homogenized, and pooled. The glucose levels were estimated in the pooled mixture of the zebrafish.

### 3.10. Histopathology Studies

Histological analysis of treated and untreated zebrafish was carried out as described by the previously established methods with minor modifications [28]. Zebrafish from the control and the treatment groups were taken and anaesthetized using 1% tricaine mesylate [29]. The anaesthetized zebrafish were fixed overnight in 1% formaldehyde and dehydrated using 80%, 90%, 95%, 100% ethanol, and 100% xylene. Dehydrated samples were embedded in paraffin and subjected to histology analysis [30,31,32,33]. Samples were sectioned and stained with haematoxylin and eosin, and the ocular regions were focused under a microscope.

### 3.11. RT-PCR

The total RNA was extracted from the ocular region of the zebrafish, utilizing the Trizol method and synthesizing the cDNA. Light Cycler 480 (Roche Applied Science, Basel, Switzerland) with SYBR Premix ExTaq (Takara, Dalian, China) were used to perform reactions in a 10 μL volume comprising 1 μL of cDNA, 5 μL of SYBR green master mix, 1 μL of a primer mix (*Vegf165*: Forward, 5′-CTC CTC CAT CTG TCT GCT GTA AAG-3′ and Reverse, 5′-CTC TCT GAG CAA GGC TCA CAG-3′; and internal control β-actin: Forward, 5′-GCC ACC TTA AAT GGC CTA GCA-3′; Reverse 5′-GCC ATA CAG AGC AGA AGC CA-3′), and 3 μL of RNase-free water per sample [7].

### 3.12. Statistics

The data presented in this study are the mean of three replicates ± standard deviation (SD). Furthermore, the obtained data were analysed on one-way ANOVA, Tukey’s comparisons test on GraphPad prism software (version 5.03) LA Jolla, USA. 

## 4. Discussion

VEGF is a key gene involved in the aetiology of DR. To be more specific, prolonged over-expression of VEGF could result in visual impairment that causes blood–retinal barrier breakdown of the retinal vessels [7]. A better understanding of treating DR would be intervening upstream to eliminate or significantly mitigate VEGF overexpression. Oxidative stress plays a major primary event in DR [1]. High oxidative stress levels increased over VEGF expressions have been reported earlier in retinal models [34,35]. Conversely, treatment strategies that diminish oxidative stress reduce VEGF expression. Therefore, in this study, we tested the activity of NG on inhibiting oxidative stress-induced cellular damage and lipid peroxidation via high glucose treatment on developing zebrafish embryos and further evaluated the effect of oxidative stress-induced VEGF expression in adult wild-type zebrafish and its morphological alterations.

Immersion of adult or larval zebrafish in glucose causes diabetes, similar to those seen in streptozotocin (STZ)-induced diabetic mice and diabetic people [3,34,35]. Furthermore, adult zebrafish retinas subjected to hyperglycaemia for 30 days show morphological alterations such as vessel thickening, loss of inter-endothelial cell–cell junction integrity, and vessel basement membrane thickening. Therefore, we treated the developing zebrafish embryos in 130 mM Glucose to induce abnormal glucose metabolism, mimicking hyperglycaemic conditions, and co-exposed the embryos with various concentrations of NG to screen the activity of NG in the zebrafish larvae. The embryos were exposed for a period from 3 hpf to 5 dpf. The embryonic exposure revealed that NG was not toxic to the embryos and showed no toxic effects on the hatching and survival rates of the embryo compared to the high glucose-induced group, where a significant difference was observed.

Glucose causes retinal damage in the diabetic condition through recurrent acute and cumulative alterations that can cause tissue destruction [36]. The retina contains the most polyunsaturated fatty acids and has the highest oxygen absorption and glucose oxidation [37]. This process makes the retina more vulnerable to oxidative stress. It has been proposed that the relationship between hyperglycaemia, redox homeostasis alterations, and oxidative stress is crucial in developing diabetic retinopathy. Therefore, when stained with DCFH-DA, high fluorescent intensity was observed in the ocular region due to high levels of ROS induced by glucose treatment, whereas the co-exposure of NG reduced the levels of ROS in a dose-dependent manner, almost similar to the control group.

Oxidative stress promotes NADPH oxidase in various retinal cells in DR, including endothelial cells, pericytes, and Müller cells. The increased activity of NADPH oxidase in response to hyperglycaemia leads to the generation of superoxide anions. A high concentration of glucose treatment induced the production of superoxide anions [38,39]. The excess glucose during diabetic conditions is necessitated to degrade in the Tricarboxylic acid cycle (TCA); consequently, additional NADH or FADH_2_ is forced into the mitochondrial electron transport chain and forces the proton gradient to reach the inner membrane to the maximum threshold. In this state, electron transport is hampered, and electrons are transferred to molecular oxygen, producing excess superoxide. The superoxide anions were stained using DHE stain, where high levels of superoxide radicals were observed in the ocular region, whereas, in the treatment group, the superoxide radicals were eliminated at higher doses of NG. 

GSH is an intracellular thiol that eliminates free radicals. GSH levels fall due to a battle between aldose reductase and glutathione reductase for NADPH, a cofactor, and increasing oxidative stress [40]. As a result of oxidative stress induced by the glucose treatment, depleted GSH levels were observed in the model group compared to the control. At the same time, the GSH levels were restored due to NG exposure. NG at high concentrations almost restored the GSH levels, preventing GSH depletion.

As a consequence of high ROS levels, these effects lead to vascular dysfunction, resulting in cellular damage and apoptosis. As the progress of the DR basement membrane thickens, pericytes and endothelial cells undergo faster cellular damage apoptosis [41,42]. The total cellular damage in the ocular region was stained using Acridine orange. The results revealed that NG inhibited cellular damage significantly in the model group, where high levels of cellular damage indicated by fluorescent intensity were observed.

Excess production of ROS leads to lipid peroxidation, which is the primary reaction of ferroptosis and is produced by an oxidative attack on lipids. Uncontrolled lipid peroxidation causes membrane rupture and cell death due to forming free radicals and oxidation products [37,41,43]. The lipid peroxidation levels were accessed using the DPPP stain, in which high levels of lipoxidation were found in the Glucose exposure group, whereas the co-exposure of NG inhibited the lipoxidation process by preventing cellular damage and lipoxidation. An optimum concentration of 75 µM NG was selected for the subsequent experiments [10].

Adult wild-type zebrafish were employed to study further the morphological alterations induced by glucose exposure. Post glucose and NG exposure, high glucose levels were found in the model group compared to the control group, with lesser glucose concentration in the NG treatment group. During diabetic complications, damage to the eyes begins when high glucose clogs the tiny blood vessels that lead to the retina, causing them to leak fluid or bleed [1]. Similarly, the histopathology analysis revealed that NG inhibited macular degeneration and necrosis in the ocular region of the zebrafish. NG at 75 µM inhibited the destruction caused by the glucose treatment.

VEGF is an important factor involved in vascular proliferation [43,44]. Anti-VEGF drugs are being studied extensively, and it is observed that DR can be regressed with VEGF treatment. Therefore, we studied the expression of the key gene VEGF in the lens of the zebrafish, where NG exposure downregulated the expression of VEGF compared to the model group. The regressed VEGF expression could prove that NG inhibited macular degeneration in adult zebrafish by downregulating VEGF. Thus, the study demonstrated that NG, which has antidiabetic, antioxidant, and antiapoptotic capabilities, may limit macular degeneration by providing neurotrophic support to minimize retinal damage in diabetic retinopathy.

## 5. Limitation

One limitation of acutely induced hyperglycemia as a model for diabetes and DR is that it does not accurately represent the chronic nature of the disease. Diabetes and DR typically develop and progress over several years, with long-term exposure to high blood glucose levels leading to vascular damage and other complications in humans. In contrast, acute induction of hyperglycemia artificially raises blood glucose levels for a short period, which may not fully capture the complex and progressive nature of the disease.

## 6. Conclusions

NG, abundantly found in citrus fruits, is known for its pharmacological effects. This study concludes the antidiabetic property of NG towards DR. NG reduced the total glucose levels in the developing embryos and improved the hatching and mortality rates. NG prevented oxidative stress and superoxide anion production in the ocular region and restored the depleted glutathione levels in the developing zebrafish larvae. When a high glucose concentration was exposed to adult wild-type zebrafish, NG inhibited the increase in glucose levels and prevented cellular damage, inhibiting macular degeneration in the retina. NG also decreased the overexpression of VEGF, which correlated with the other results. Therefore, an ophthalmic intervention of NG could be an efficient source of drugs for preventing DR. However, this study is limited by animal models and could be further evaluated in a mammalian model before human trials.

## Data Availability

The data that support the findings of this study are available on request from the corresponding author.
